# MicroRNA-1229 overexpression promotes cell proliferation and tumorigenicity and activates Wnt/β-catenin signaling in breast cancer

**DOI:** 10.18632/oncotarget.8119

**Published:** 2016-03-16

**Authors:** Zhanyao Tan, Haiqing Zheng, Xiangxia Liu, Wenhui Zhang, Jinrong Zhu, Geyan Wu, Lixue Cao, Junwei Song, Shu Wu, Libing Song, Jun Li

**Affiliations:** ^1^Program of cancer research, Affiliated Guangzhou Women and Children's Hospital, Zhongshan School of Medicine, Sun Yat-Sen University; ^2^State Key Laboratory of Oncology in Southern China, Department of Experimental Research, Sun Yat-sen University Cancer Center; ^3^Department of Rehabilitation Medicine, The third Affiliated Hospital, Sun Yat-sen University, Guangzhou, China; ^4^Department of Plastic Surgery, The First Affiliated Hospital of Sun Yat-sen University; ^5^Department of Pathology, The First Affiliated Hospital of Sun Yat-sen University

**Keywords:** MiR-1229, breast cancer, proliferation, Wnt/β-catenin pathway

## Abstract

Constitutive activation of the Wnt/β-catenin pathway promotes malignant proliferation and it is inversely correlated with the prognosis of patients with breast cancer. However, mutations in key regulators, such as APC, Axin and β-catenin, contribute to aberrant activation of the Wnt/β-catenin signaling pathway in various cancers, but rarely found in breast cancer, suggesting that other mechanisms might be involved in the activation of Wnt/β-catenin signaling in breast cancer. In the present study, we found that miR-1229 expression was markedly upregulated in breast cancer and associated with poor survival. Overexpressing miR-1229 promoted while inhibiting miR-1229 reduced, proliferation of breast cancer cell proliferation *in vitro* and tumor growth *in vivo*. Furthermore, we found that overexpression of miR-1229 activated the Wnt/β-catenin signaling pathway in breast cancer by directly targeting the multiple important negative regulators of Wnt/β-catenin signaling, including adenomatous polyposis coli (APC), glycogen synthase kinase-3β (GSK-3β), and inhibitor of β-catenin and T cell factor (ICAT). Taken together, our results suggest that miR-1229 plays an important role in promotion breast cancer progression and may represent a novel therapeutic target in breast cancer.

## INTRODUCTION

Breast cancer is the second most frequent cancer in the world and represents the leading type of cancer in women [1]. Approximately 1.68 million new cases occur and 522,000 people die of breast cancer worldwide [1]. Despite better surgery, cytotoxic agents, and endocrine therapy achieving significant progress in the optimization of treatment, the prognosis for patients with breast cancer has not improved much [2-5]. Accumulating evidence indicates that aberrant activation of Wnt/β-catenin signaling promotes cell proliferation and survival and enhances characteristics of the malignant phenotype in breast cancer and is associated with poor prognosis of breast cancer patients [6-9], suggesting that targeting Wnt/β-catenin signaling might be a potential strategy for breast cancer treatment. However, the mechanisms leading to aberrant activation of Wnt/β-catenin signaling in breast cancer remain largely elusive.

In the canonical Wnt/β-catenin signaling, when Wnt ligands engage the Frizzled receptor and low-density lipoprotein receptor-related protein-5/6 (LRP5/6), Dishevelled (Dvl) is phosphorylated by Frizzled and leads to disruption of destruction complex formed by glycogen synthase kinase-3β(GSK-3β), adenomatosis polyposis coli (APC), Axin, protein phosphatase 2A (PP2A) and casein kinase 1α (CK1α). Consequently, β-catenin translocates into the nucleus to bind transcriptional factors of the T cell factor/lymphoid enhancer binding factor (TCF/LEF) family, resulting in transcription of multiple downstream genes involved in cell proliferation [6, 10, 11].

Multiple research groups have demonstrated that constitutive activation of the canonical Wnt pathway in cancer cells is due to loss-of-function or deletion mutations in key negative factors in Wnt/β-catenin signaling, such as APC, Axin, or β-catenin [12]. It has been reported that defects in the APC gene lead to inherited and sporadic forms of colon cancer and may account for up to 80% of the cancers in colon tissue [13]. Meanwhile, approximately 10% of hepatic cancer contains Axin2 mutations [12]. In addition, β-catenin mutations have been found in 26% of primary pancreatic tumors [14]. However, the current data indicate that these mutations are extremely rare in breast cancer [15], suggesting that other mechanisms are involved in activation of the β-catenin signaling pathway in breast cancer.

MicroRNAs (miRNAs), a class of endogenous noncoding small RNAs, are involved in regulating a wide range of crucial biological processes, including differentiation, apoptosis and proliferation [16]. It has been demonstrated that miRNAs play vital roles in regulating cell proliferation in breast cancer [17-20]. Previously, it has been reported that miR-1229 expression was significantly increased in coronary artery calcification (CAC) patients plasma and was correlated with the degree of CAC [21]. Constantly, the levels of miR-1229 were significantly higher in serum exosomes in patients with primary colorectal cancer (CRC), even those with early stage disease, than in healthy control, indicating that miR-1229 might play important role in CRC progression and may serves as a promising biomarker for diagnosis of colon cancer [22].

In the current study, we reported that miR-1229 was overexpressed in breast cancer and correlated with poor prognosis for patients with breast cancer. MiR-1229 upregulation promoted cell proliferation in breast cancer both *in vitro* and *in vivo*, and activated Wnt/β-catenin signaling by directly targeting GSK-3β, APC, and inhibitor of β-catenin and T cell factor (ICAT). Therefore, our results uncover a novel mechanism in which Wnt/β-catenin signaling is constitutively activated in breast cancer and represent a potential target for breast cancer therapy.

## RESULTS

### MiR-1229 was upregulated in breast cancer and associated with poor prognosis

**Figure 1:**
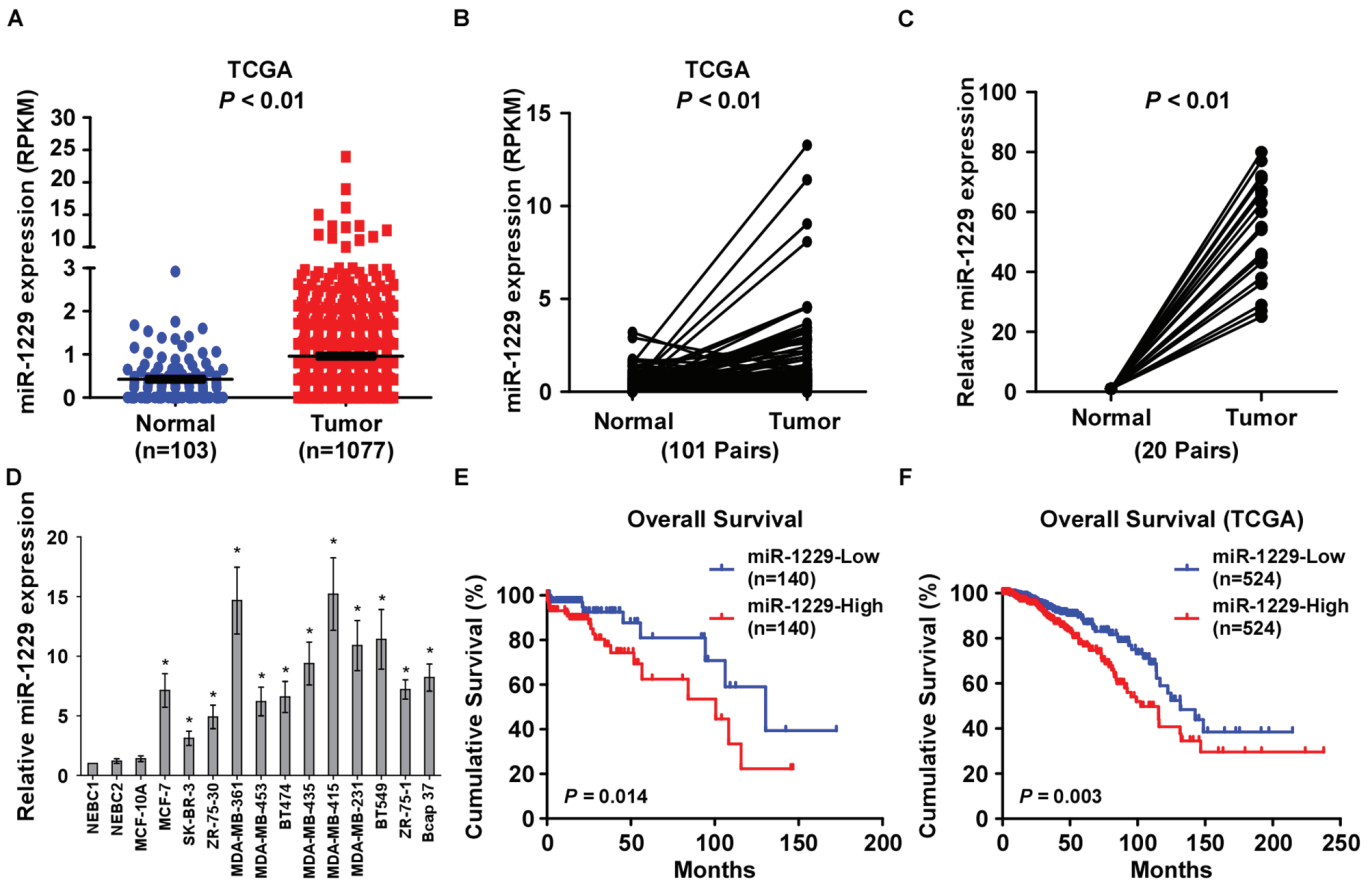
MiR-1229 is upregulated in breast cancer tissues and cell lines and is associated with poor prognosis **A.** Expression profile of miR-1229 in primary breast cancer tissues (n = 1077) and normal breast tissues (n = 103) (*P* < 0.01; TCGA). **B.** Relative expression of miR-1229 in 101 paired primary breast cancer tissues (Tumor) and normal breast tissue (*P* < 0.01; TCGA). **C.** Real-time PCR analysis of miR-1229 expression in primary breast cancer tissues (Tumor) with matched adjacent normal breast tissue (Normal) from 20 paired patients (*P* < 0.01). **D.** Relative miR-1229 expression in 12 breast cancer cell lines, immortalized MCF-10A normal breast epithelial cells, and two NBEC lines. MiRNA levels were normalized to that of *U6* RNA. Bars represent the mean ± SD of three independent experiments (*P < 0.01). **E.** Kaplan–Meier analysis of overall survival stratified by low miR-1229 expression (<median, n = 140) and high miR-1229 expression (>median, n = 140). MiR-1229 upregulation was significantly correlated with shorter overall survival (*P =* 0.014). **F.** Kaplan–Meier analysis of overall survival stratified by low miR-1229 expression (n = 524, TCGA, blue) and high miR-1229 expression (n = 524, TCGA, red) (*P =* 0.003).

By analyzing the miRNA sequencing datasets from The Cancer Genome Atlas (TCGA), we found that miR-1229 was significantly upregulated in primary breast cancer tissues compared with normal breast tissue (breast cancer : n = 1077, normal: n=103; *P* < 0.05; Figure 1A) and 101 primary breast cancer tissues compared with corresponding paired normal breast tissue (Figure 1B). Furthermore, real-time PCR analysis showed that miR-1229 expression was upregulated in 20 primary breast cancer tissues as compared with matched adjacent normal breast tissues, and in 12 breast cancer cell lines compared with that in normal immortalized MCF-10A breast cell line and in two normal breast epithelial cell (NBEC) lines (Figure 1C-1D). Therefore, the published miRNA datasets and our results suggest that miR-1229 is upregulated in breast cancer.

To further investigate whether upregulated miR-1229 is involved in breast cancer progression, the correlation between miR-1229 levels and the clinical pathological features of breast cancer (n=280) was examined. Statistical analyses showed that miR-1229 expression was positively correlated with clinical stage (*P* < 0.001), and TNM classification (T: *P* < 0.001, N: *P* < 0.001, M: *P =* 0.002) (Supplementary Table S2). Additionally, Kaplan–Meier survival analysis revealed that breast cancer patients with higher expression of miR-1229 had shorter overall survival, which further confirmed by the results obtained from TCGA data (Figure 1E-1F). Moreover, univariate and multivariate analyses indicated that miR-1229 was recognized as an independent prognostic factor in breast cancer (Supplementary Table S3). These results indicate a possible link between miR-1229 upregulation and breast cancer progression.

### MiR-1229 upregulation promoted breast cancer cell proliferation *in vitro*

**Figure 2:**
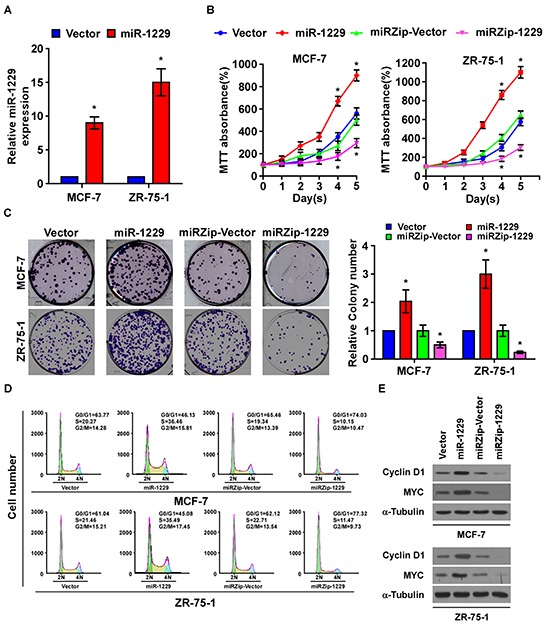
MiR-1229 upregulation promotes breast cancer cell proliferation *in vitro* **A.** Real-time PCR analysis of miR-1229 expression in the indicated cells. MiRNA levels were normalized to that of U6 RNA. **B.** MTT assay revealing that miR-1229 upregulation promoted MCF-7 and ZR-75-1 cell growth. **C.** Representative micrographs (left) and quantification (right) of crystal violet–stained cell colonies. **D.** Flow cytometric analysis of MCF-7 and ZR-75-1 cells. **E.** Western blotting analysis of cyclin D1 and c-MYC expression in the indicated cells. α-Tubulin served as the loading control. Bars represent the mean ± SD of three independent experiments.

To determine the effect of miR-1229 overexpression on breast cancer progression, we established MCF-7 and ZR-75-1 breast cancer cell lines that stably overexpressed miR-1229 (Figure 2A). As shown in Figure 2B and 2C, overexpressing miR-1229 dramatically promoted the growth rate of the MCF-7 and ZR-75-1 cells *in vitro* as analyzed by tetrazolium (MTT) and colony formation assays. Furthermore, flow cytometry assay showed that miR-1229 overexpression significantly increased the percentage of breast cancer cells in the S phase and decreased the percentage of cells in the G0/G1 phase (Figure 2D). Consistently, downregulation of miR-1229 drastically inhibited cell proliferation *in vitro* and resulted in G1/S arrest (Figure 2B-2D). Moreover, overexpressing miR-1229 upregulated, while miR-1229 knockdown downregulated, the expression of cyclin D1 and MYC. Taken together, these results suggest that miR-1229 overexpression induces proliferation *in vitro* and promotes G1/S transition of breast cancer cells.

### Overexpression of miR-1229 enhanced tumorigenicity of breast cancer cells

**Figure 3:**
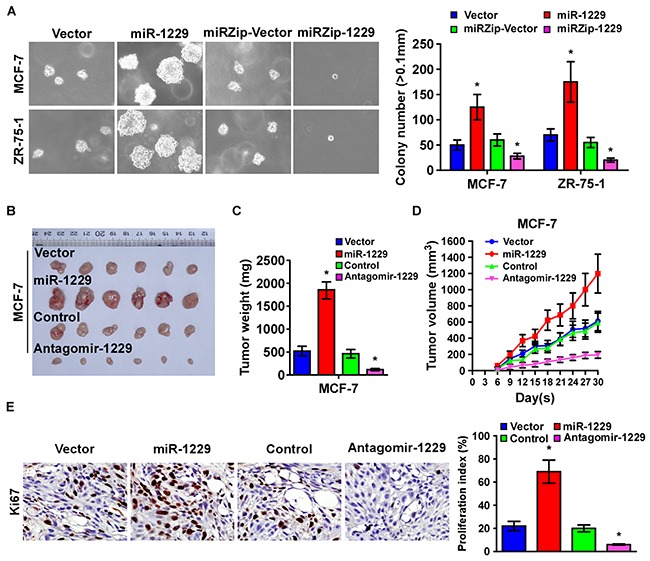
Silenced endogenous miR-1229 inhibits breast cancer tumorigenicity *in vitro* and tumor growth *in vivo* **A.** Representative images (left) and quantification (right) of cell colony numbers as determined by anchorage-independent growth assay. Colonies > 0.1 mm in diameter were scored. **B.** Tumors formed by indicated cells in nude mice (n = 6/group). **C.** Histograms of the mean tumor weights of each group. **D.** Tumor formation growth curves after implantation of indicated cells. **E.** IHC staining showing that miR-1229 upregulation increased the percentages of Ki-67–positive cells in the tumors, whereas miR-1229 downregulation inhibited the percentages of Ki-67 positive cells in the tumors. Bars represent the mean ± SD of three independent experiments. *P < 0.05.

To determine the oncogenic role of miR-1229 in tumorigenicity of breast cancer cells *in vitro*, we conducted an anchorage-independent growth ability assay. As shown in Figure 3A, miR-1229 overexpression drastically increased, but downregulation of miR-1229 decreased, the anchorage-independent growth ability of the MCF-7 and ZR-75-1 cells. To test whether upregulation of miR-1229 could enhance breast cancer tumor growth *in vivo*, miR-1229–overexpressed and vector control cells were inoculated into nude mice. After 4 weeks, the tumors formed by the miR-1229–overexpressing MCF-7 cells were larger and heavier than the control tumors, but the tumors formed by MCF-7 cells treated with antagomir-1229 were smaller and lighter than the control tumors (Figure 3B–3D). Furthermore, immunohistochemical (IHC) analysis revealed that, compared to the control tumors, miR-1229–overexpressing tumors had higher percentages of Ki-67–positive cells, whereas miR-1229–silenced tumors had lower percentages of Ki-67–positive cells (Figure 3E). Collectively, these results indicate that miR-1229 overexpression promotes tumorigenicity *in vitro* and tumor growth *in vivo*.

### MiR-1229 activated Wnt/β-catenin signaling by targeting multiple negative regulators

**Figure 4:**
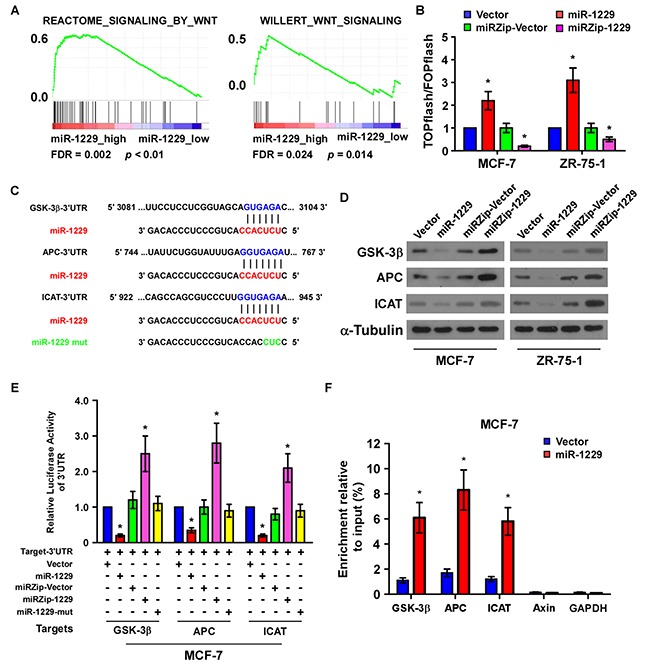
MiR-1229 activates the Wnt/β-catenin signaling pathway by targeting GSK-3β, APC, and ICAT **A.** GSEA plot showing that miR-1229 expression was positively correlated with Wnt/β-catenin target gene signatures (REACTOME SIGNALING BY WNT and WILLERT_WNT_SIGNALING) in a published breast cancer dataset (TCGA). **B.** TOPflash or FOPflash luciferase reporter activity analyzed in the indicated cells. Cells transfected with TOPflash or FOPflash and *Renilla* pRL-TK plasmids were subjected to dual-luciferase assays 48 hours after transfection. The reporter activity detected was normalized by *Renilla* luciferase activity. **C.** The predicted miR-1229 target sequence in the 3′UTR of *GSK3B*, *APC*, and *ICAT*. The miR-1229 mutant (miR-1229-mut) contained three altered nucleotides in the seed sequence. **D.** Western blotting of GSK-3β, APC, and ICAT expression in MCF-7 and ZR-75-1 cells. α-Tubulin was used as the loading control. **E.** Luciferase assay of the indicated cells transfected with pGL-3UTR reporter and Vector, miR-1229, miRZip-Vector, miRZip-1229, or miR-1229-mut. **F.** RIP assay revealing the selective association between miR-1229 and *GSK-3β, APC, or ICAT*. *Axin and GAPDH* served as negative control. Bars represent the mean ± SD of three independent experiments. *P < 0.05.

Gene set enrichment analysis (GSEA) revealed significantly upregulated Wnt/β-catenin signaling gene sets in the patients expressing high levels of miR-1229 (Figure 4A), suggesting that miR-1229 upregulation may activate Wnt/β-catenin signaling. As expected, β-catenin activity was significantly increased in the miR-1229–overexpressing cells but was decreased in the miR-1229–silenced cells (Figure 4B). Concordantly, the expression levels of multiple downstream targets of Wnt/β-catenin signaling, namely cyclin D1, c-MYC, TCF4 and LEF1, and nuclear β-catenin, were increased in the miR-1229–overexpressing breast cancer cells but decreased in the miR-1229–silenced breast cancer cells (Supplementary Figure S1A–S1C). Taken together, these results suggest that miR-1229 overexpression promotes the activation of Wnt/β-catenin signaling in breast cancer.

**Figure 5:**
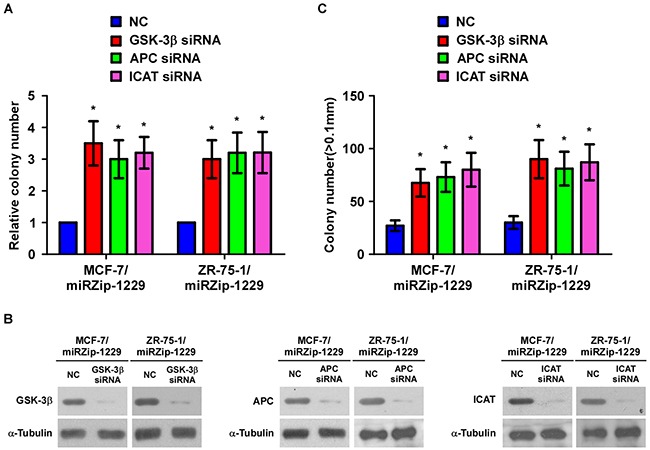
Suppression of GSK-3β, APC, and ICAT is functionally important for the biological effects of miR-1229 in breast cancer cells **A.** Quantification of crystal violet–stained breast cancer cell colonies formed after 10 days. **B.** Western blotting analysis of GSK-3β, APC, and ICAT expression levels in miR-1229–silenced cells transfected with siRNA against GSK-3β, APC, or ICAT. α-Tubulin served as the loading control. **C.** Quantification of cell colony numbers as determined by anchorage-independent growth assay. Colonies > 0.1 mm in diameter were scored. Each bar represents the mean ± SD of three independent experiments. *P < 0.05.

Publically available algorithms (miRanda, TargetScan) were used to explore the precise mechanism by which miR-1229 activates the Wnt/β-catenin pathway in breast cancer, and showed that GSK-3β, APC, and ICAT might be potential targets of miR-1229 (Figure 4C). As predicted, western blotting revealed that expression of GSK-3β, APC, and ICAT was decreased in miR-1229–overexpressing MCF-7 and ZR-75-1 cells but was increased in miR-1229–inhibited MCF-7 and ZR-75-1 cells (Figure 4D). Luciferase reporter analysis showed that miR-1229 overexpression reduced, but downregulation of miR-1229 increased, the luciferase reporter activity of the 3′ untranslated region (3′UTR) of GSK-3β (*GSK3B*), *APC* and *ICAT*. However, overexpression of miR-1229-mut did not affect the luciferase reporter activity of the 3′UTR of these three genes (Figure 4E). Furthermore, the RNA immunoprecipitation (RIP) assay revealed that miR-1229 only specifically associated with the 3′UTR of *GSK3B*, *APC*, and *ICAT*, but not with that of *AXIN* and glyceraldehyde-3-phosphate dehydrogenase (*GAPDH*) (Figure 4F). These results suggest that miR-1229 activates the Wnt/β-catenin signaling pathway by targeting GSK-3β, APC, and ICAT. Moreover, targeting these three negative regulators promoted the proliferation of miR-1229–silenced breast cancer cells. Consistently, we also found that miR-1229 levels were significantly correlated with nuclear β-catenin expression but inversely associated with the levels of GSK-3β, APC and ICAT in mice tumors formed with miR-1229-dysregulated cells (Supplementary Figure S2). Importantly, individual silencing of GSK-3β, APC, or ICAT dramatically increased the rate of cell proliferation and growth (Figure 5A–5C), further demonstrating that GSK-3β, APC, and ICAT are essential effectors of miR-1229–induced breast cancer cell proliferation.

### MiR-1229 levels correlated with Wnt/β-catenin signaling activation in breast cancer

**Figure 6:**
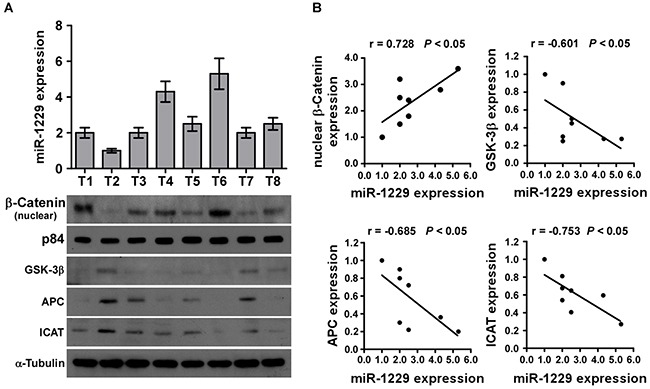
MiR-1229 levels correlate with activation of Wnt/β-catenin signaling, β-catenin nuclear accumulation, and GSK-3β, APC, and ICAT expression in clinical breast cancer clinical tissues **A.** Real-time PCR of miR-1229 expression and western blotting of nuclear b-catenin, GSK-3β, APC, and ICAT expression in eight fresh breast cancer tissue specimens (T). U6 was used as the RNA loading control, p84 was used as nuclear control; α-tubulin was used as the protein loading control. **B.** Correlation between miR-1229 levels and GSK-3β, APC, ICAT, and nuclear β-catenin expression in clinical breast cancer tissues. The expression levels of GSK-3β, APC, ICAT and β-catenin, checked by western blotting analysis, were determined by densitometry. The ratio of second sample (GSK-3β/α-tubulin, APC /α-tubulin, ICAT/α-tubulin and β-catenin /α-tubulin) was considered as 1.0.

To examine whether miR-1229 levels correlate with activation of Wnt/β-catenin signaling and GSK-3β, APC, and ICAT expression in clinical breast cancer tissue, real-time PCR was used to detect miR-1229 expression in eight fresh breast cancer tissues and western blotting was performed to detect GSK-3β, APC, and ICAT expression. As shown in Figure 6A and 6B, there was a significant inverse correlation between miR-1229 and GSK-3β (r = −0.601; *P* < 0.05), APC (r = −0.685; *P* < 0.05), and ICAT (r = −0.753; *P* < 0.05) expression but a significant positive correlation between miR-1229 and nuclear β-catenin expression (r = 0.728; *P* < 0.05). Taken together, these results further support the premise that miR-1229 upregulation promotes proliferation by activating Wnt/β-catenin signaling in breast cancer.

## DISCUSSION

In the present study, we provided evidence for a novel mechanistic link between miR-1229 and the oncogenic Wnt/β-catenin signaling in breast cancer. We found that miR-1229 was significantly upregulated in breast cancer and was inversely correlated with poor prognosis. Overexpression of miR-1229 promoted, while downregulation of miR-1229 inhibited, breast cancer cell proliferation both *in vitro* and *in vivo*. Furthermore, we demonstrated that miR-1229 activated the Wnt/β-catenin pathway by directly targeting GSK-3β, APC, and ICAT, the vital negative regulators of the Wnt/β-catenin pathway. Taken together, our results indicate that miR-1229 functions as an oncomiR in breast cancer and may represent an important target for clinical intervention in breast cancer.

Aberrant activation of the Wnt/β-catenin signaling pathway is commonly found in many types of human cancer and is thought to promote tumor progression [23-28]. Multiple key negative regulators, such as GSK-3β, APC, and ICAT, have been found to be suppressed in cancer, which contributes to the promotion of tumor progression through regulation of the oncogenic Wnt/β-catenin pathway. For instance, Carotenuto et al. showed that h-prune interaction with GSK-3β, which impairs the ability of GSK-3β to phosphorylate β-catenin, activates the Wnt/β-catenin pathway and enhances cancer progression in non–small cell lung cancer (NSCLC) [29]. Kinase inactivation of GSK-3β, such as by the GSK-3β inhibitor AR79, promotes prostate cancer growth and mammary tumorigenesis by activating the canonical Wnt pathway [30, 31]. On the other hand, loss of APC function in mouse models was found to lead to hyperactivation of Wnt/β-catenin signaling and results in colorectal tumorigenesis [32]. Gaspar et al. reported that mice with a heterozygous truncated APC mutant had elevated Wnt/β-catenin signaling activity and developed mammary adenocarcinomas and subsequent pulmonary metastases [33]. Meanwhile, dowmregulation of negative regulation of ICAT could promote glioma tumorigenesis by activating the Wnt/β-catenin signaling pathway [34]. Herein, we demonstrated that miR-1229 simultaneously suppressed GSK-3β, APC, and ICAT expression by directly targeting their 3′UTR. Consistent with the tumor-suppressive effects of GSK-3β, APC, and ICAT, miR-1229 was upregulated in breast cancer, and its overexpression dramatically promoted breast cancer cell proliferation both *in vitro* and *in vivo*. Taken together, our results represent a novel mechanism of GSK-3β, APC, and ICAT downregulation in breast cancer and a functionally and clinically relevant epigenetic mechanism of breast cancer pathogenesis. Interestingly, it has been reported that the levels of miR-1229 was significantly higher in serum exosomes in patients with primary colorectal cancer, even those with early stage disease, than in healthy controls, suggesting that miR-1229 might be a promising biomarker for diagnosis of colon cancer [22]. Meanwhile, via assessment of a published microarray, we also found that the expression of miR-1229 was also significantly upregulated in multiple cancers (Supplementary Figure S3), which further supported the notion that miR-1229 might function as an important oncomiR and might contribute to multiple cancer progression.

The Wnt/β-catenin signaling pathway is controlled at multiple levels. For example, the indicator of Wnt signaling activation, β-catenin, is degraded by a destruction complex formed by GSK-3β, APC, Axin, and CK1α[10, 35] and its interaction with TCF is negatively regulated by ICAT [36]. In addition, Wnt/β-catenin signaling pathway is also regulated by β-catenin subcellular localization. Krieghoff et al. reported that APC slows down β-catenin nucleo-cytoplasmic shuttling and thereby retains it in the cytoplasm [37]. Herein, we demonstrated that miR-1229 suppressed the Wnt signaling negative regulators GSK-3β, APC, and ICAT by targeting their 3′UTR, thus enhancing cytoplasm-to-nuclear transition and transcriptional activity of β-catenin, suggesting that that miR-1229 might activate the Wnt/β-catenin signaling pathway through multiple mechanisms via inhibiting β-catenin degradation, accumulating nuclear β-catenin levels and increasing β-catenin transcriptional activity.

Kinase inhibitors have become preferred for anti-tumors [38]. Due to their significant impact on tumor progression, anti-tumor drugs targeting the Wnt/β-catenin signaling pathway have aroused considerable worldwide interest [38, 39]. However, the two key kinases in Wnt signaling, GSK-3β and CK1α, negatively regulate the Wnt/β-catenin signaling pathway, and therefore cannot serve as therapeutic targets for anti-tumor treatment. Thus, discovering novel molecule(s) that play important roles in inactivation of the Wnt/β-catenin signaling pathway will be of great clinical potential for treating cancer. In this study, miR-1229 activated Wnt/β-catenin signaling by targeting three key negative regulators. Importantly, inhibiting miR-1229 via the miRZIP system or downregulating miR-1229 via its antagomiR significantly decreased cell proliferation *in vitro* and tumor growth *in vivo* in breast cancer. These findings suggest that small molecule inhibitors of miR-1229 might have therapeutic potential against breast cancer.

In summary, the present study demonstrated that miR-1229 promoted tumor growth in breast cancer. MiR-1229 upregulation drastically promoted breast cancer cell proliferation by inhibiting the expression of three key negative regulators (GSK-3β, APC, and ICAT) of the Wnt/β-catenin signaling pathway. These findings uncover a novel mechanism of Wnt/β-catenin signaling pathway hyperactivation in breast cancer, and miR-1229 may serve as a potential therapeutic target in breast cancer.

## MATERIALS AND METHODS

### Cell lines and cell culture

Human breast cancer cell lines (MCF-7, SK-BR-3, ZR-75-30, MDA-MB-361, MDA-MB-453, BT474, MDA-MB-435, MDA-MB-415, MDA-MB-231, BT549, ZR-75-1 and Bcap 37) and immortalized normal breast epithelial cells (MCF-10A) were purchased from American Type Culture Collection (ATCC, Manassas, VA, USA). All cancerous cell lines were grown in Dulbecco's modified Eagle's medium (DMEM; Invitrogen, Carlsbad, CA, USA) supplemented with 10% fetal bovine serum (FBS; Invitrogen). Primary normal breast epithelial cells (NBECs) and immortal breast cell line MCF-10A were cultured in keratinocyte serum free medium (KSFM) supplemented with 0.1 ng/mL human recombinant epidermal growth factor and 20 μg/mL bovine pituitary extract (Invitrogen). All cell lines were maintained in a humidified incubator with 5% CO2 at 37°C.

### Patients and tumor tissues

This study was conducted on a total of 280 paraffin embedded breast cancer samples and 20 human breast cancer tissues with matched adjacent normal breast tissues, which were histopathologically and clinically diagnosed at the Sun Yat-sen University Cancer Center between 2003 and 2010. For the clinical materials used in our study, prior patient consent and approval from the Institutional Research Ethics Committee (IREC) were obtained. Clinical and clinicopathologic classification and stage were determined according to the American Joint Committee on Cancer (AJCC) criteria. Clinical information on the samples is shown in Supplementary Table S1.

### RNA extraction, reverse transcription, and real-time PCR

Total RNA from tissues or cells was extracted using TRIzol (Life Technologies) according to the manufacturer's instructions. Messenger RNA (mRNA) and miRNA were polyadenylated using a poly-A polymerase-based First-Strand Synthesis kit (TaKaRa Bio, DaLian, China) and reverse transcription (RT) of total mRNA was performed using a PrimeScript RT Reagent kit (TaKaRa) according to the manufacturer's protocol. Complementary DNA (cDNA) was amplified and quantified on ABI 7500HT system (Applied Biosystems, Foster City, CA, USA) using SYBR Green I (Roche, Grenzach-Wyhlen, Germany). Supplemental Information lists the primers used in the reactions. Primers for U6 and miR-1229 were synthesized and purified by RiboBio (Guangzhou, China). U6 or glyceraldehyde-3-phosphate dehydrogenase (GAPDH) was used as endogenous controls. Relative fold expressions were calculated with the comparative threshold cycle (2^−ΔΔCt^) method.

### Plasmid, small interfering RNA and transfection

The human miR-1229 gene was PCR-amplified from genomic DNA and cloned into a pMSCV-puro retroviral vector (Clontech, Tokyo, Japan). MiRZIP was constructed by System Biosciences. Transfection of plasmids was performed using Lipofectamine 2000 (Life Technologies) according to the manufacturer's instructions. The reporter plasmids containing wild-type (CCTTTGATC; TOPflash) or mutated (CCTTTGGCC; FOPflash) TCF/LEF DNA binding sites were purchased from Upstate Biotechnology. The partial 3'-untranslated region (3'-UTR) region (containing miR-1229 target site) of the human GSK-3β, APC, and ICAT was PCR-amplified from genomic DNA and cloned into pGL3 vectors (Promega, Madison, WI, US), and the plasmid phRL-tk was used as the internal control for transfection efficiency and cytotoxicity of test chemicals (Promega). A list of primers used in the reactions was presented in Supplemental Information. AntagomiR-1229 was purchased from RIBOBIO Company (Guangzhou, China).

### Xenografted tumor model, H&E, and IHC staining

All experimental procedures were approved by the Institutional Animal Care and Use Committee of Sun Yat-Sen University and housed in barrier facilities on a 12 h light/dark cycle. BALB/c-nu mice (4-5 weeks of age, 18-20 g) were purchased from the Center of Experimental Animal of Guangzhou University of Chinese Medicine. The BALB/c nude mice were randomly divided into groups (n = 6/group). One group of mice was inoculated subcutaneously with MCF-7/Vector cells (2 × 10^6^) in the left dorsal flank and with MCF-7/miR-1229 cells (2 × 10^6^) in the right dorsal flank per mouse. Another two groups were inoculated subcutaneously per mouse with MCF-7 cells (2 × 10^6^) in both dorsal flank. 7 days later, the mice inoculated MCF-7 cells were then intratumorally injected with one hundred microliters of antagomir control or antagomiR-1229 (diluted in PBS at 2 mg/ml) 3 times per week for 2 weeks. Tumors were examined twice weekly; length, width, and thickness measurements were obtained with calipers and tumor volumes were calculated. Tumor volume was calculated using the equation *volume (mm^3^) =* (L × W^2^)/2. On day 30, tumors were detected by an IVIS imagining system (Caliper), then animals were euthanized, tumors were excised and subjected to pathologic examination.

### 3-(4, 5-Dimethyl-2-thiazolyl)-2, 5-diphenyl-2H-tetrazolium bromide (MTT) assay

Cells (2 × 10^3^) were seeded into 96 well plates and stained at the indicated time point with 100μl sterile 3-(4,5-dimethythiazol-2-yl)-2,5-diphenyl tetrazolium bromide (MTT; Sigma-Aldrich, St Louis, MO) dye (at 0.5 mg/ml) for 4 h at 37°C, followed by removal of the culture medium and the addition of 150μl dimethyl sulfoxide (Sigma-Aldrich). The absorbance was measured at 570 nm, with 655 nm used as the reference wavelength.

### Colony formation assay

Cells (0.2 × 10^3^) were plated into six well plates and cultured for 10 days. Colonies were then fixed for 5 min with 10% formaldehyde and stained with 1.0% crystal violet for 30s.

### Anchorage-independent growth ability (soft agar) assay

Cells (3 × 10^3^) were suspended in 2 ml complete medium plus 0.3% agar (Sigma-Aldrich). The agar–cell mixture was plated as a top layer onto a bottom layer comprising 1% complete medium agar mixture. After 10 days culture, colony size was measured using an ocular micrometer and colonies >0.1 mm in diameter were counted.

### Flow cytometry analysis

Cells (5 × 10^5^) were harvested by trypsinization, washed in ice-cold phosphate-buffered saline and fixed in 80% ice-cold ethanol in phosphate-buffered saline (PBS). Before staining, cells were gently sedimented and resuspended in cold PBS. Bovine pancreatic ribonuclease (Sigma-Aldrich) was added to a final concentration of 2 μg/ml, and cells were incubated at 37°C for 30 min, followed by incubation with 20 μg/ml propidium iodide (Sigma-Aldrich) for 20 min at room temperature. Cell samples (2 × 10^4^) were then analyzed by Gallios flow cytometer (Beckman Coulter, Brea, CA, USA) and the data were analyzed using FlowJo 7.6 software (TreeStar Inc., Ashland, OR, USA).

### Western blotting analysis

Cell lysates were separated by 10% sodium dodecyl sulfate–polyacrylamide gel electrophoresis and transferred to polyvinylidene fluoride membranes (Millipore, Billerica, MA, USA). The membranes were probed with antibodies against Cyclin D1, MYC, TCF4, LEF1, β-catenin, GSK-3β, APC, and ICAT (Abcam, Cambridge, MA) overnight at 4°C, and then incubated with horseradish peroxidase–conjugated secondary antibodies (Cell Signaling Technology) for 1 h at room temperature. The blotting membranes were stripped and re-probed with an anti-α-Tubulin antibody (Sigma, Saint Louis, MO).

### Luciferase reporter assay

Cells were plated in 100-mm cell culture dishes, proliferating to 60–80% confluence after 24 h of culture. The reporter constructs were transfected using Lipofectamine 2000 (Life Technologies) according to the manufacturer's protocol. After 12-h incubation, the transfection medium was replaced; cells were harvested and washed with PBS, and lysed with passive lysis buffer (Promega). The cell lysates were analyzed immediately using a 96-well plate luminometer (Berthold Detection System, Pforzheim, Germany). Luciferase and Renilla luciferase were measured using a Dual-Luciferase Reporter Assay System (Promega) according to the manufacturer's instructions. The luciferase activity of each lysate was normalized to Renilla luciferase activity. The relative transcriptional activity was converted into fold induction above the vehicle control value.

### RNA immunoprecipitation (RIP)

Cells were cotransfected with pMSCV-miR-1229 or pMSCV-vector and pIRESneo-FLAG/HA-Ago2 expression vector (Addgene plasmid 10822; Addgene Inc.). After 48-h transfection, cells were washed and lysed in RNA immunoprecipitation buffer (Sigma-Aldrich) containing 10% proteinase inhibitor cocktail (Sigma-Aldrich) and 1mM phenylmethylsulfonyl fluoride (Sigma-Aldrich). A fraction of the whole cell lysate was used for RNA isolation, and the remaining lysate was subjected to immunoprecipitation (IP) using an antibody against Ago2 (Abcam). RNA from whole cell lysates and RNA IP (RIP) fractions was extracted with TRIzol (invitrogen) according to the manufacturer's instructions. The relative levels of GSK-3β, APC, and ICAT, Axin and GAPDH mRNA were determined using real-time RT-PCR as described above. The relative mRNA enrichment in the RIP fractions was computed based on the ratio of relative mRNA levels in the RIP fractions and the relative mRNA levels in the whole cell lysates (input).

### Statistical analysis

All values were presented as means ± standard deviation (SD). Significant differences were determined using SPSS 16.0 software (SPSS, Chicago, IL, USA). Student's t-test was used to determine statistical differences. The chi-square test was used to analyze the relationship between miR-1229 expression and clinical pathological characteristics. Survival curves were plotted using the Kaplan Meier method and compared by log-rank test. *P* < 0.05 was considered significant.

## SUPPLEMENTARY MATERIALS AND METHODS, FIGURES AND TABLES


