# Supplementary material for: MicroRNA-1229 overexpression promotes cell proliferation and tumorigenicity and activates Wnt/β-catenin signaling in breast cancer
Source: Oncotarget. 2016 Mar 16;7(17):24076–87. doi: 10.18632/oncotarget.8119 (PMC5029685; doi:10.18632/oncotarget.8119)
Supplement: Supplementary file 1 [file oncotarget-07-24076-s001.pdf]

SUPPLEMENTARY MATERIALS AND METHODS

Primers used for real-time RT-PCR

GAPDH-up	TTGAGGTCAATGAAGGGGTC
GAPDH-dn	GAAGGTGAAGGTCGGAGTCA
Cyclin D1-up	GGCGGATTGGAAATGAACTT
Cyclin D1-dn	TCCTCTCCAAAATGCCAGAG
MYC-up	CACCGAGTCGTAGTCGAGGT
MYC-dn	TTTCGGGTAGTGGAAAACCA
TCF4-up	TCTCCATAGTTCCTGGACGG
TCF4-dn	CCAACTTCTTTGGCAAGTGG
LEF1-up	CACTGTAAGTGATGAGGGGG
LEF1-dn	TGGATCTCTTTCTCCACCCA

Primers used for subcloning and plasmid construction:

miR-1229-up	CTAGGCGCCGGAATTAGATCTCTTAGTCGGCCTTTTTCAGGAA
miR-1229-dn	GTAGAATTCGTTAACCTCGAGCTCACGTTCTTCAGTTTCCGC
pGL3- GSK-3β-up	TAATTCTAGAGCTCCCCGCGGCCTTTTTCTGTGCTGATGGGTT
pGL3- GSK-3β-dn	CCCCGACTCTAGAAACTGCAGAACAACAGTCCTCTATTGGCACG
pGL3- APC -up	TAATTCTAGAGCTCCCCGCGGTATTTAAAGTAGCATCCCATCCCA
pGL3- APC -dn	CCCCGACTCTAGAAACTGCAGATAGATAAGTGCCAACGCATGGA
pGL3-ICAT-up	TAATTCTAGAGCTCCCCGCGGTACATTTGCTTTAATGAGTGCACCT
pGL3-ICAT-dn	CCCCGACTCTAGAAACTGCAGTTCATAAACAAACGATACTGTCCCTT

SUPPLEMENTARY FIGURES AND TABLES

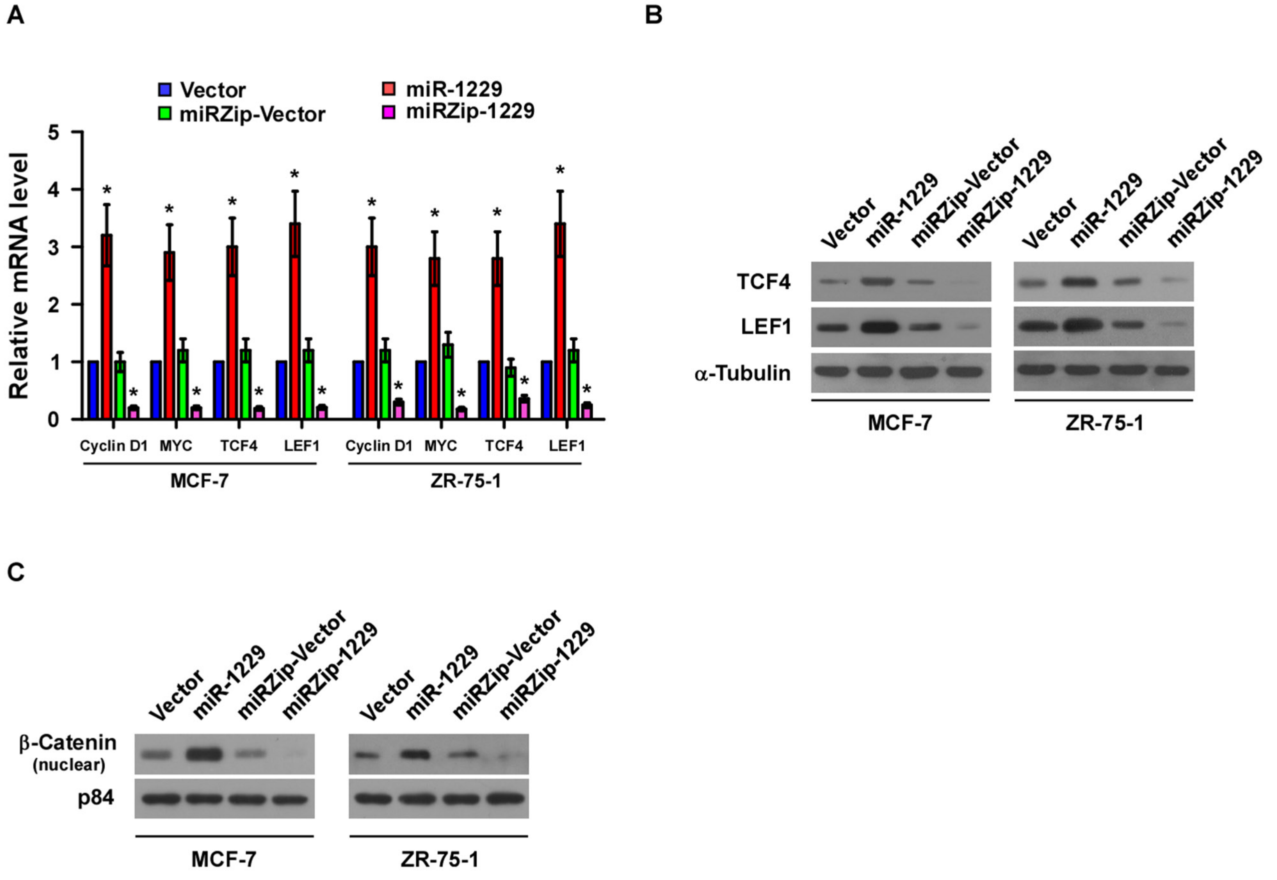

Supplementary Figure S1: miR-1229 activates Wnt/β-catenin signaling pathway. A. Real-time analysis of Cyclin D1, MYC, TCF4 and LEF1 mRNA expression in MCF-7 and ZR-75-1 cells. GAPDH served as the loading control. Error bars represent the mean ± SD from three independent experiments. * $P < 0.05$. **B.** Western blotting analysis of TCF4 and LEF1 in MCF-7 and ZR-75-1 cells. α-Tubulin served as the loading control. **C.** Western blotting analysis of nuclear β-catenin expression in the indicated cells. p84 served as the loading control.

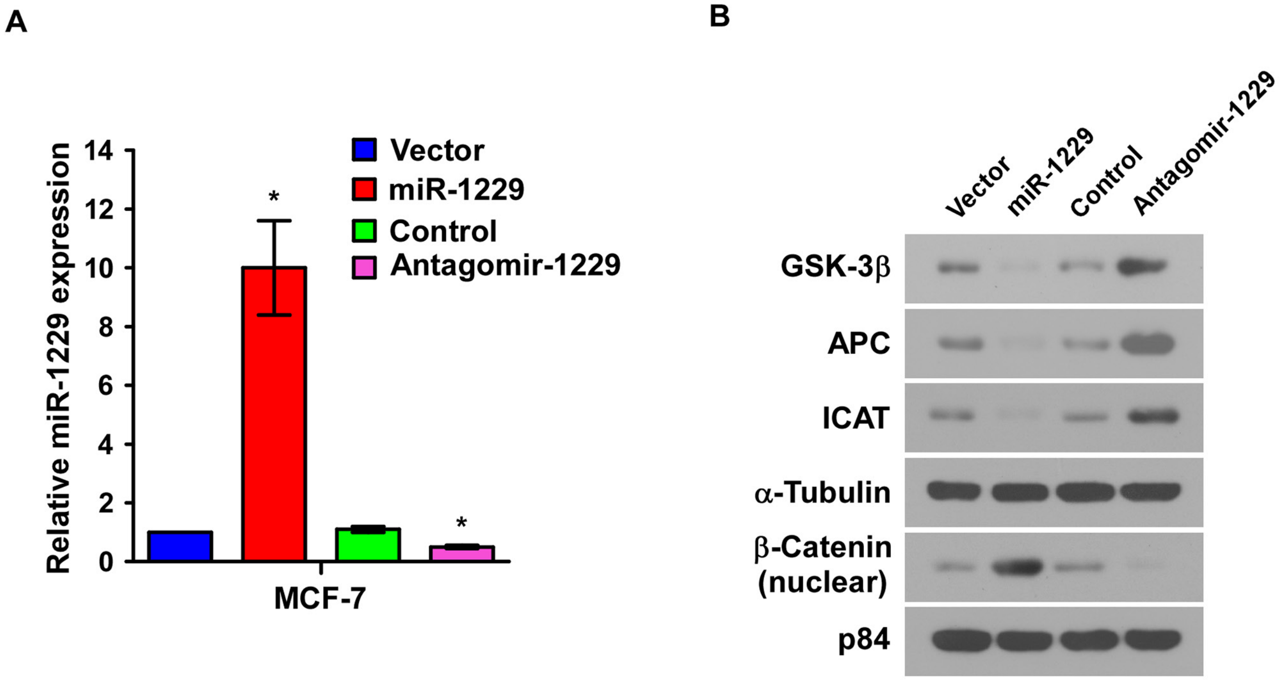

Supplementary Figure S2: MiR-1229 activates Wnt/β-catenin signaling pathway by downregulation of GSK-3β, APC, and ICAT. A. Real-time PCR analysis of miR-1229 in the indicated xenografts tumors. *$P < 0.05$. **B.** Western blot analysis of the expression levels of GSK-3β, APC, and ICAT expression, and nuclear β-Catenin in indicated xenograft tumors.

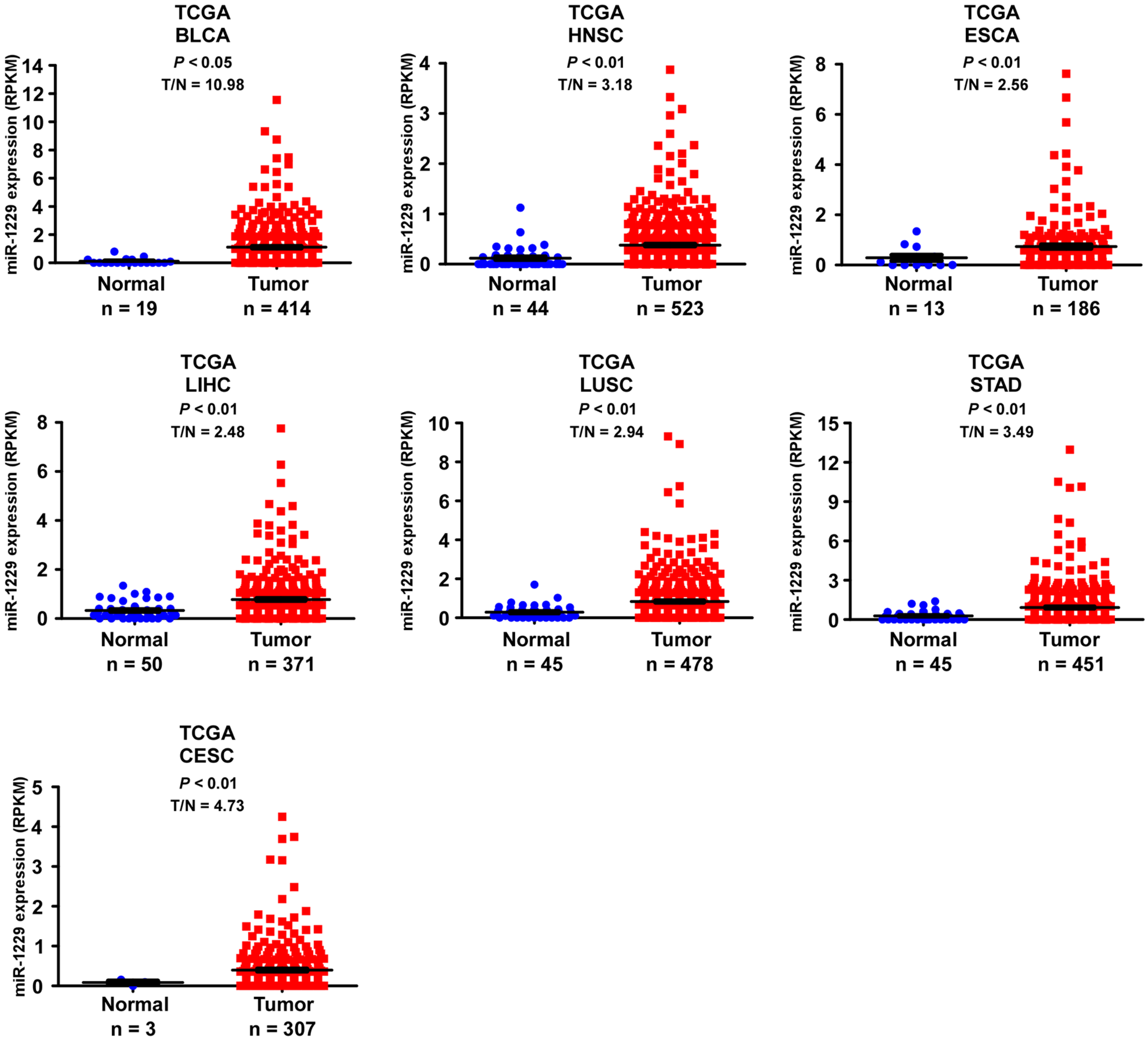

Supplementary Figure S3: The expression of miR-1229 was upregulated in multiple human cancer types (TCGA).

Supplementary Table S1: Clinicopathological characteristics of studied patients and expression of miR-1229 in breast cancer

Factor	NO.	(%)
**Age (years)**		
≤53	95	33.9
>53	185	66.1
**Clinical stage**		
I	34	12.2
II	132	47.1
III	79	28.2
IV	35	12.5
**T classification**		
T_1	53	18.9
T_2	150	53.6
T_3	58	20.7
T_4	19	6.8
**N classification**		
N_0	120	42.9
N_1	107	38.1
N_2	43	15.4
N_3	10	3.6
**M classification**		
No	270	96.4
Yes	10	3.6
**ER**		
Negative	131	46.8
Positive	149	53.2
**PR**		
Negative	119	42.5
Positive	161	57.5
**HER2**		
Negative	58	20.7
Positive	222	79.3
**Expression of miR-1229**		
Low expression	140	50.0
High expression	140	50.0

Supplementary Table S2: Correlation between the clinicopathological features and expression of miR-1229

Patient characteristics		MiR-1229 expression		*P*-value
		Low or none	High	
**Age (years)**	≤50	51	44	0.449
	>50	89	96	
**Clinical stage**	I	25	9	< 0.001
	II	74	58	
	III	36	43	
	IV	5	30	
**T classification**	T_1	35	18	< 0.001
	T_2	77	73	
	T_3	25	33	
	T_4	3	16	
**N classification**	N_0	74	46	< 0.001
	N_1	51	56	
	N_2	13	30	
	N_3	2	8	
**M classification**	No	140	130	0.002
	Yes	0	10	
**ER**	Negative	77	54	0.008
	Positive	63	86	
**PR**	Negative	71	48	0.008
	Positive	69	92	
**HER2**	Negative	23	35	0.104
	Positive	117	105	

Supplementary Table S3: Univariate and multivariate analysis of different prognostic parameters in patients with breast cancer by Cox-regression analysis

	Univariate analysis		Multivariate analysis	
	**P**	**Hazard ratio (95% CI)**	**P**	**Hazard ratio-RR (95% CI)**
**Clinical stage**				
I				
II	0.001	0.399 (0.229-0.692)	0.114	0.526 (0.237-1.167)
III				
IV				
**T classification**				
T_1				
T_2	< 0.001	0.334 (0.187-0.597)	0.029	0.363 (0.147-0.900)
T_3				
T_4				
**N classification**				
N_0				
N_1	0.005	0.506 (0.313-0.816)	0.261	0.644 (0.299-1.387)
N_2				
N_3				
**MiR-1229 expression**				
Low expression	0.017	2.466 (1.172-5.190)	< 0.001	7.181 (3.040-16.965)
High expression				
